# Crystallographic and Spectroscopic Investigations on Oxidative Coordination in the Heteroleptic Mononuclear Complex of Cerium and Benzoxazine Dimer

**DOI:** 10.3390/molecules26175410

**Published:** 2021-09-06

**Authors:** Worawat Wattanathana, Natapol Suetrong, Peetikamol Kongsamai, Kantapat Chansaenpak, Nutthawat Chuanopparat, Yuranan Hanlumyuang, Pongsakorn Kanjanaboos, Suttipong Wannapaiboon

**Affiliations:** 1 Department of Materials Engineering, Faculty of Engineering, Kasetsart University, Ladyao, Chatuchak, Bangkok 10900, Thailand; natapol.s@ku.th (N.S.); yuranan.h@ku.th (Y.H.); 2 School of Chemistry, Institute of Science, Suranaree University of Technology, 111 University Avenue, Suranaree, Muang, Nakhon Ratchasima 30000, Thailand; peetikamol.k@gmail.com; 3 National Nanotechnology Center, National Science and Technology Development Agency, Thailand Science Park, Khlong Luang 12120, Thailand; kantapat.cha@nanotec.or.th; 4 Department of Chemistry, Faculty of Science, Kasetsart University, Ladyao, Chatuchak, Bangkok 10900, Thailand; fscinwc@ku.ac.th; 5 School of Materials Science and Innovation, Faculty of Science, Mahidol University, Nakhon Pathom 73170, Thailand; pongsakorn.kan@mahidol.edu; 6 Synchrotron Light Research Institute, 111 University Avenue, Suranaree, Muang, Nakhon Ratchasima 30000, Thailand

**Keywords:** crystal structure, cerium complex, benzoxazine, benzoxazine dimer, X-ray absorption spectroscopy

## Abstract

Among lanthanide-based compounds, cerium compounds exhibit a significant role in a variety of research fields due to their distinct tetravalency, high economic feasibility, and high stability of Ce(IV) complexes. Herein, a systematic investigation of crystallographic information, chemical properties, and mechanistic formation of the novel Ce(IV) complex synthesized from cerium(III) nitrate hexahydrate and 2,2′-(methylazanediyl)bis(methylene)bis(4-methylphenol) (MMD) ligand has been explored. According to the analysis of the crystallographic information, the obtained complex crystal consists of the Ce(IV) center coordinated with two nitrate ligands and two bidentate coordinated (*N*-protonated and O,O-deprotonated) MMD ligands. The fingerprint plots and the Hirshfeld surface analyses suggest that the C–H⋯O and C–H⋯π interactions significantly contribute to the crystal packing. The C–H⋯O and C–H⋯π contacts link the molecules into infinite molecular chains propagating along the [100] and [010] directions. Synchrotron powder X-ray diffraction (XRD) and X-ray absorption spectroscopy (XAS) techniques have been employed to gain an understanding of the oxidative complexation of Ce(IV)-MMD complex in detail. This finding would provide the possibility to systematically control the synthetic parameters and wisely design the precursor components in order to achieve the desired properties of novel materials for specific applications.

## 1. Introduction

Cerium compounds exhibit a significant role in various research fields ranging from organic synthesis, inorganic–organic hybrid materials, catalysis, materials science, and renewable energy applications [1,2,3,4]. Among lanthanide-based compounds, Ce is the only element that forms distinct tetravalent molecular complexes and exhibits the redox-active interconversion between +3 and +4 oxidation states [5]. Moreover, cerium precursor sources are economically feasible with respect to the other lanthanide sources. A variety of Ce(IV) complexes can be collected due to the high stability of the Ce(IV) ion possessing [Xe] electronic configuration [6]. These results indicate the possibility to explore their reactivities along with physicochemical characteristics systematically. Interestingly, a multitude of structures and chemical functionalities of coordinated ligands, as well as coordination environments around Ce ions, lead to the fine-tuning of the local electronic environment around the Ce center, resulting in a variety of redox potentials, catalytic activities, and optical properties [4,7]. Such emerging photoluminescent complexes [8,9] have drawn attention for applications in light-emitting materials, photosensitizers for photocatalysis, and photovoltaic devices. The systematic investigation of structural variations and the correlated physicochemical properties of the Ce-based complexes would provide an opportunity to achieve the design of novel materials, which are suitable for the aforementioned applications.

At a closer inspection into the preparation of Ce(IV) complexes, there are two main synthetic approaches to obtain the desired materials, namely the ligand substitution reactions of Ce(IV) precursors and the one-electron oxidation of Ce(III) complexes [4]. Considering the ligand substitution approach, CeO_2_, [CeCl_6_]^2−^, and ceric ammonium nitrate (CAN) are commonly used as the starting precursors [10,11]. However, the ligands possessing a reducing property may disturb the preservation of the desired tetravalent oxidation state [12]. Therefore, the second approach to get the Ce(IV) complexes via the oxidation of Ce(III) precursors has become more mature. According to the literature, three mechanistic reaction types of the Ce(III) oxidation reactions have been developed to form stable Ce(IV) complexes, namely (a) oxidative functionalization, (b) outer-sphere oxidation and auto-oxidation reactions, and (c) oxidation-induced ligand redistribution reactions. The oxidative functionalization is the simultaneous inner-sphere oxidation of the Ce center together with the insertion of new ligands to the coordination sphere, such as the use of PhICl_2_ to oxidize the Ce(III) complex: Ce[N(SiMe_3_)_2_]_3_ and later the insertion of the Cl^−^ ligand to form the Ce(IV) complex: ClCe[N(SiMe_3_)_2_]_3_ [13]. For the outer-sphere oxidation and auto-oxidation reaction, atmospheric oxygen [14,15,16] and/or metal-salt oxidants [17,18] are applied to oxidize the Ce(III) precursors, e.g., cerium(III) chloride [14] and cerium(III) acetylacetonate [15] during the introduction of ligands to form the desired Ce(IV) complexes. In the oxidation-induced ligand redistributions, the reactions take place in Ce(III) precursors under oxidizing (or aerobic) environments to generate the Ce(IV) products [19,20]. It is generally accepted that the designs of ligands to be applicable as good donors, which lower the oxidation potential of Ce into the range of general oxidative synthetic conditions, are crucial.

Dihydro-1,3,*2H*-benzoxazines formed by a one-pot Mannich reaction of phenols, primary amines, and formaldehyde [21,22] are one promising class of ligands possessing structural diversity. Dihydro-1,3,*2H*-benzoxazines are also called “benzoxazine monomers” due to the possibility to be used as starting precursors for the ring-opening polymerization in order to prepare polybenzoxazines [23,24,25,26]. Interestingly, using phenol as an initiator to perform the ring-opening reaction of benzoxazine monomers does not form polybenzoxazines. Instead, the formations of intramolecular hydrogen bonds in the obtained products terminate the progressive polymerization reaction, which consequently lead to the formation of “dihydro-benzoxazine dimer” molecules possessing the aza-bis(methylene)-bis(phenol) moiety [27]. Dihydro-benzoxazine dimers (shortly called benzoxazine dimers) resemble the repeating unit in polybenzoxazines, which can be used as simplified models for studying the properties of polybenzoxazines [28]. Benzoxazine dimers provide one nitrogen and two oxygen donor atoms which can bind to tremendously diversified metal ions in various coordination geometries ranging from monodentate, bidentate, and tridentate coordination modes [29,30]. Hence, benzoxazine dimers exhibit promising properties to be utilized in various applications such as catalysis [31,32,33], optical luminescence [34], pollutant removal [35], and medical applications [36,37]. Due to the interesting characteristics of benzoxazine dimers, their use as chelating ligands for Ce-based complexes has attracted attention and is worth investigating in detail.

In the previous work [30], benzoxazine dimers were used as chelating ligands to form a complex with Ce(III) salt. The main goal was to use the obtained product as a single-source precursor for the synthesis of the pure CeO_2_ phase via the thermal decomposition method. In this work, we extend the scope of study towards a systematic investigation of crystallographic information, physicochemical properties, and mechanistic formation of the desired Ce-benzoxazine dimer complex, which could further provide a thorough understanding to offer the rational molecular designs of such novel materials for a variety of applications. Not only the well-controlled single-source precursors for mixed-metal oxide syntheses, but also the applications for redox catalysis, photocatalysis, and photoluminescence can be further achieved from metal-benzoxazine dimer compounds. Herein, the complexation between cerium(III) nitrate hexahydrate and the benzoxazine dimer so-called 2,2′-(methylazanediyl)bis(methylene)bis(4-methylphenol) (MMD) [38] is examined and used as a model system to expand the understanding of other benzoxazine dimer derivatives. Interestingly, the single crystal of the Ce(IV)-MMD complex is obtained even though the Ce(III) precursor is used. The detailed investigation of crystallographic information is discussed herein, which reveals the driving force for the formation of the stable Ce(IV)-MMD compound. In addition, synchrotron powder X-ray diffraction (XRD) and X-ray absorption spectroscopy (XAS) coupled with chronoamperometry have been employed to examine the oxidative complexation formation of Ce(IV)-MMD.

## 2. Materials and Methods

### 2.1. Chemicals

Paraformaldehyde was purchased from Sigma (St. Louis, MO, USA), while p-cresol, methylamine (40% *w*/*v* in water), and sodium sulfate anhydrous were bought from Fluka Chemicals (Buchs, Switzerland). Cerium (III) nitrate hexahydrate (Ce(NO_3_)_3_⋅6H_2_O, 99.5% purity) was acquired from Acros Organics, while sodium hydroxide was obtained from Ajax Fine chemicals. The organic solvents, i.e., ethanol, dioxane, dichloromethane, diethyl ether, and propan-2-ol were supplied from RCI Labscan. All chemicals were analytical grade and used as received.

### 2.2. Preparation of the Ligand (MMD)

The compound 2,2′-(methylazanediyl)bis(methylene)bis(4-methylphenol) (MMD), also called herein a benzoxazine dimer, is prepared by a two-step process. Firstly, the benzoxazine monomer (3,4-dihydro-3,6-dimethyl-1,3,*2H*-benzoxazine) was synthesized by a Mannich reaction using p-cresol, formaldehyde, and methylamine with the molar ratio of about 1:2:1 as the starting precursors [38]. Note that a slight excess amount of formaldehyde and methylamine was used to allow all p-cresol to be completely reacted. Paraformaldehyde (9.9 g, or 330 mmol) was dissolved in dioxane (60 mL) in a round bottom flask, and then methylamine (15.6 mL, or 180 mmol) was added to the flask. The reaction mixture was stirred for 15 min, and subsequently p-cresol (16.2 g, or 150 mmol) was added. Then, the reaction mixture was refluxed for 6 h. After the reaction was finished, the obtained product was extracted by dichloromethane (70 mL), washed with 3N sodium hydroxide, and then washed with deionized water. The extracted product was dried using anhydrous sodium sulfate. The solvent was further removed by evaporation under reduced pressure to obtain the viscous yellow liquid of the prepared benzoxazine monomer.

To obtain the desired MMD ligand, the equimolar amount of p-cresol was then mixed with the obtained benzoxazine monomer to carry out the ring-opening dimerization. The reaction was conducted in a neat state by heating the mixture at 60 °C for a night. The crude product was washed with diethyl ether and then re-crystallized in propan-2-ol for purification.

### 2.3. Preparation and Characterization of the Cerium-MMD Complex

The cerium-MMD complex (Ce-MMD) was obtained by mixing the 0.02 M ethanolic solutions of cerium(III) nitrate hexahydrate and the MMD ligand with the molar ratio of 1:2. Immediately after mixing the precursors, the color of the mixture solution was changed from colorless to brown, suggesting a complex formation. After that, the mixture was left in contact with an ambient atmosphere for 2 days, and some amounts of the solvent were allowed to be evaporated. As a result, the dark brown single crystals of Ce-MMD were formed.

The single-crystal X-ray diffraction data were collected on a Bruker APEXII CCD diffractometer controlled by APEXII software, and the cell refinement and data reduction were carried out by *SAINT* [39]. Absorption correction was conducted by the multi-scan method using *SADABS* [40]. The structure was solved by SHELXT program [41]. The structure was refined on F^2^ by a full-matrix least-squares method using SHELXL software [42]. All non-hydrogen atoms were treated anisotropically, while the H atoms were treated by riding on the ideal positions. Software packages used to prepare molecular graphics and materials for publication were Olex2 [43] and Mercury [44]. 

To study the chemical functionalities, FTIR spectra were collected by Bruker Alpha FTIR spectrometer. The samples were ground and uniaxially pressed with potassium bromide (spectroscopic grade) into pellets before measurements. The one hundred scans of the samples and the backgrounds were taken in the wavenumber range of 4000–375 cm^−1^. The spectral resolution was ±2 cm^−1^. In addition, FT Raman spectra were recorded by the Fourier transform Raman spectrophotometer (Horiba, LabRAM HR Evolution model) using a red laser with a wavelength of 785 nm in the range of 2100–300 cm^−1^. The acquisition time per step was set to 5 s, and the spectral data were the average value from 10 accumulated scans. To examine the structural changes during the complexation, a nuclear magnetic resonance spectrometer (Bruker AVANCE III 400 MHz) was used to measure the ^1^H NMR spectra of the MMD ligand and the cerium-MMD complex. The samples were dissolved in CDCl_3_ prior to the NMR investigation. The elemental analysis of the Ce-MMD complex was carried out using a LECO CHNS/O analyzer. Thermal aspects of the samples were determined by a differential scanning calorimeter (DSC 1 STAR^e^ System, Metler Toledo, Columbus, OH, USA). The samples were weighed and placed in the aluminum pans prior to the DSC measurement. The samples were heated from −80 °C to 300 °C under the flow of air. The heating rate was set to 5 °C/min. Moreover, the weight losses of the samples were studied by thermogravimetric analysis (TGA) using a Shimadzu 60 AH TG/DTG analyzer performed in an air zero atmosphere with the temperature range from 25 °C to 1000 °C. The heating rate for the TGA measurement was 5 °C/min.

^1^H NMR: (MMD, 400 MHz, CDCl_3_) δ 8.80 (s, 2H, OH), 7.15–6.86 (m, 4H, H aromatic), 6.76 (dt, *J* = 13.2, 6.5 Hz, 2H, H aromatic), 3.74 (s, 4H, CH_2_N), 2.37 (2xs, 9H, CH_3_N and CH_3_ aromatic). 

^1^H NMR: (the cerium-MMD complex, 400 MHz, CDCl3) δ 10.22 (s, 2H, NH), 7.07 (*d*, *J* = 8.3 Hz, 4H, H aromatic), 6.92 (*d*, *J* = 8.3 Hz, 4H, H aromatic), 6.83 (s, 4H, H aromatic), 5.00–4.87 (m, 4H, CH_2_N), 3.75–3.57 (m, 4H, CH_2_N), 2.60 (s, 6H, CH_3_N), 2.34 (s, 12H, CH_3_ aromatic). Anal. calc. for C_34_H_40_CeN_4_O_10_: C, 50.74%; H, 5.01%; N, 6.96%; O, 19.88%. Found: C, 50.72%; H, 4.99%; N, 6.94%; O, 19.89%.

To understand the oxidative complexation of Ce-MMD, synchrotron powder X-ray diffraction (XRD) and X-ray absorption spectroscopy (XAS) were carried out at Beamline 1.1W: Multiple X-ray Techniques (MXT), Synchrotron Light Research Institute (Public Organization), Thailand. In addition to the MMD ligand and the Ce-MMD crystal, the crude product was prepared by evaporating the ethanolic solution mixture between the cerium(III) nitrate hexahydrate and the MMD ligand under reduced pressure (hereafter, named as “Ce-MMD crude”). XRD patterns of the MMD ligand, the Ce-MMD crude, and the Ce-MMD crystal were collected in Debye–Scherrer geometry using a monochromatic synchrotron X-ray with an energy of 12 keV (wavelength of 1.0332 Å) and the strip detector (Mythen6K 450, DECTRIS^®^). The fine powder samples were packed into a Kapton capillary (diameter of 0.5 mm). The capillary sample was attached to the goniometer head and adjusted to the right position. Then, the continuous spinning of the capillary was carried out during the measurement.

To monitor the oxidative complexation of Ce-MMD, the ethanolic solution mixture between the cerium(III) nitrate hexahydrate and the MMD ligand (named as “Ce-MMD solution”) was applied with a fixed voltage of 1 V by performing chronoamperometry using an EmStat3/3+ Blue Potentiostat controlled by the PSTrace 5.8 software (PalmSens, Houten, The Netherlands). Note that a conventional three-electrode system including a glassy carbon working electrode (diameter 3 mm), a Pt wire as a counter electrode, and an Ag/Ag+ (0.01 M AgNO_3_) reference electrode was used for applying the voltage to the mixture. The obtained solutions and/or the precipitated powders after applying a static voltage of 1 V for 0.5 h, 1 h, 1.5 h, 2 h, 4 h, and 6 h were collected. In this case, a notation of “Ce-MMD-1V-xh”, where x is the time (h) used for applying a voltage to the solution, was used. After that, the XAS probed at Ce L3 edge in the X-ray absorption near edge structure (XANES) region was carried out at an ambient temperature and pressure to identify the chemical speciation. The spontaneous measurements of both transmission mode and fluorescence mode were employed using an ionization chamber as the detector for monitoring the intensity of the incident X-ray before, and of the transmitted X-ray after, the absorption of the sample and using a 19-element Ge detector (Canberra) for monitoring the corresponding fluorescence signal occurred after the absorption of the sample. The XANES spectra of the Ce-MMD solution, the Ce-MMD crude, the Ce-MMD crystal, the standard Ce(NO_3_)_3_, and the standard CeO_2_ were collected and compared with the obtained products from the chronoamperometry. The XANES data processing was performed using ATHENA software [45].

## 3. Results and Discussion

### 3.1. Crystal Structure and Hirshfeld Surface Analysis of the Cerium-MMD Complex

**Figure 1:**
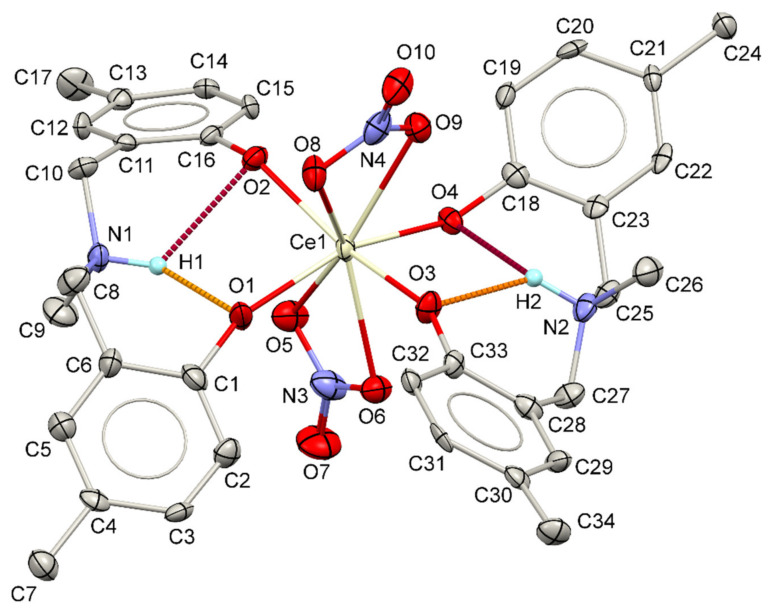
Molecular structure of the Ce-MMD complex including the non-IUPAC atomic labeling scheme. The displacement ellipsoids are drawn at the 50% probability level. The intramolecular N–H⋯O hydrogen bonds are shown as dark red and orange dash lines. Selected bond lengths (Å) and angles (°): Ce1–O1 = 2.167(5) Å; Ce1–O2 = 2.208(5) Å; Ce1–O3 = 2.175(5) Å; Ce1–O4 = 2.161(5) Å; Ce1–O5 = 2.527(5) Å; Ce1–O6 = 2.531(5) Å; Ce1–O8 = 2.557(5) Å; Ce1–O9 = 2.532(5) Å; C8–N1–C9 = 112.4(6)°; C8–N1–C10 = 113.1(6)°; C9–N1–C10 = 111.2(6)°; C25–N2–C26 = 111.6(6)°; C25–N2–C27 = 113.3(6)°; C26–N2–C27 = 110.9(6)°.

**Figure 2:**
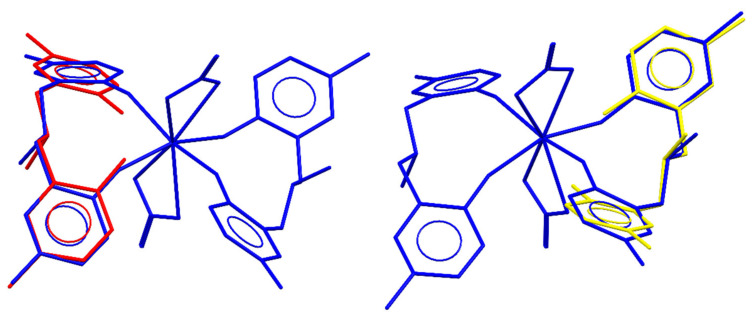
Molecular overlay diagrams of the Ce-MMD complex (blue structures) and the MMD ligand (IDUHEV from ref. [38] shown in red and yellow structures). Twenty non-hydrogen atoms of the cerium-MMD complex, namely C1–C17/N1/O1–O2, are overlayed to the same atoms in the MMD ligand (red structure) with the r.m.s. deviation of 0.394 Å. Twenty non-hydrogen atoms of the Ce-MMD complex, namely C18–C34/N2/O3–O4, are overlayed to the same atoms in the MMD ligand (yellow structure) with an r.m.s. deviation of 0.397 Å.

Complexation of the MMD ligand and cerium (III) ion takes place very rapidly, which can be identified by the abrupt change in color from colorless to brown after mixing the ethanolic MMD and cerium(III) nitrate hexahydrate solutions. Unlike the phenolic ligands without the amine moiety, the complexation of the MMD ligand does not require an alkali such as sodium hydroxide as an initiator [46]. This observation could be explained by the presence of the nitrogen atom in the MMD molecule, which can directly act as the Lewis base. Therefore, the nitrogen atom can abstract the phenolic proton and consequently convert the phenol to the phenolate group, which is more reactive to the complexation with the cerium(III) ion. After the solution was left in an ambient atmosphere for 2–3 days, the dark brown single crystals were crystallized. To obtain the crystallographic information of the obtained complex, single-crystal X-ray diffraction was carried out. The details of the crystallographic data and the corresponding structural refinement parameters are tabulated in Table 1. The molecular structure of the obtained complex (Ce-MMD complex) is depicted in Figure 1. According to Figure 1, the obtained complex is classified as the heteroleptic mononuclear complex, where the cerium ion is dative-covalently bonded by two nitrates (NO_3_^−^) and two MMD ligands. Note that the solvent molecules (ethanol) used for crystallization do not take part in both the coordination sphere and the crystal structure of the obtained complex. Interestingly, both of the MMD molecules exhibit a bidentate coordination mode to the cerium ion via two oxygen atoms of the phenolate moieties. Herein, the coordinated MMD ligand possesses two deprotonated phenolate groups (each of them has a −1 charge), while the tertiary amine nitrogen is protonated (+1 charge), forming the R_3_NH ammonium group. Therefore, the overall charge of each coordinated MMD ligand is −1. The presence of the ammonium N–H is stabilized by two *S*(6) N–H⋯O hydrogen bond motifs [47,48,49] (Table 2). Since the ammonium group is formed, the corresponding C–N–C bonds observed in the Ce-MMD complex (C8–N1–C9, C8–N1–C10, C9–N1–C10, C25–N2–C26, C25–N2–C27, C26–N2–C27) are different from the C–N–C bonds in the uncoordinated MMD ligand [38]. This is because the nitrogen atom in the pristine MMD ligand adopts the trigonal pyramidal geometry. The formation of the R_3_N ammonium salt is implied to be due to the proton transfer from the phenol moiety to the aza group, since the *S*(6) O–H⋯N intramolecular hydrogen bonds are commonly observed in the benzoxazine dimer derivatives [38,50,51,52,53,54,55,56]. Therefore, the alkali was not required for the complexation. Considering the molecular overlay diagrams between the Ce-MMD complex and the MMD ligand (IDUHEV) as shown in Figure 2, the geometrical orientations of the MMD moieties are relatively similar in both cases, suggesting the preferred bidentate coordination mode of the MMD ligand to the Ce center to form the stable Ce-complex. Insight into the Ce–O bond lengths in the Ce-MMD complex shows that the Ce–O bond lengths are pretty similar among other Ce(IV) complexes, but they are significantly shorter than the Ce–O bonds of the nitrate ligands. 

When closely inspecting the charge balance of the Ce-MMD complex, the oxidation state of cerium should be +4 to balance the total −4 charge of the ligands, namely two nitrate ligands and two *N*-protonated and O,O-deprotonated MMD ligands (singly negative charge per ligand), suggesting that the Ce(III) ion is oxidized to Ce(IV) during the complex formation. Since no additional oxidizing agent is added to the reaction mixture, the ambient oxygen in the air acts as the oxidizing agent to react with the Ce(III) ion and consequently form the Ce(IV) center of the complex. In comparison, the ethanolic solution of cerium(III) nitrate does not undergo oxidation to Ce(IV) when the solution is in contact with the ambient air, even for a long time. This information suggests that the MMD ligand plays a crucial role to promote the oxidation process of Ce(III) by lowering the oxidation potential of Ce into the range of general oxidative synthetic conditions (herein, the oxygen in ambient air). The oxidation of Ce(III) to form the Ce(IV)-MMD complex can be assigned to the outer-sphere oxidation mechanism [4,14,15,16]. The oxidative complexation formation of the Ce(IV)-MMD complex will be further discussed in detail in Section 3.3.

According to the charge neutrality of the obtained complex, the cerium ion in the complex must have the oxidation state of +4 to balance the total charge of −4 from two nitrate ligands and two *N*-protonated and O,O-deprotonated MMD ligands. In order to confirm the oxidation state of Ce(IV), the bond valence sum (BVS) method proposed by I.D. Brown [57,58,59] was done according to Equations (1) and (2). In the BVS method, the oxidation state (*OS*) is the sum of the valence, *s_ij_*, of all the *j* bonds of the metal ion *i*, *m_i_* (Equation (1)) [57,58,59]. The valence of the individual bond, *s_ij_*, can be calculated from Equation (2), where *R_ij_* is the observed bond length, and *R*_0_ is a constant value depending on the type of metal ion (*m_i_*), its oxidation state as well as the nature of the atoms bonded to the metal ion (*ij* pair). The parameter *b* is also the constant that can be found in the bond valence parameters reported by I. D. Brown [57,58,59]. For our system (the cerium complex), the *b* value was assumed to be 0.37 [57,58,59].

(1) $$OS=\;\sum_{j}s_{ij}$$

(2) $$s_{ij}=\exp\left[(R_{o}\;-\;R_{ij}\right)/b]$$

Palenik and co-workers determined the *R_0_* value for the Ce–O bonds in different cerium complexes [60]. They also extended their studies with the hundreds of the cerium complexes in the CSD database [61] and recommended the *R_0_* value for Ce(IV)–O to be 2.07 Å [62]. In our complex, we used the BVS method to calculate the oxidation state of the cerium ion in the cerium-MMD complex following Equations (1) and (2). All the bonds used in our calculation were the eight Ce–O bonds, namely Ce1–O1, Ce1–O2, Ce1–O3, Ce1–O4, Ce1–O5, Ce1–O6, Ce1–O8, and Ce1–O9, which are listed in Figure 1. The computed oxidation state was found to be +3.68 (also seen from the CheckCIF report), confirming the oxidation state of Ce(IV). 

**Figure 3:**
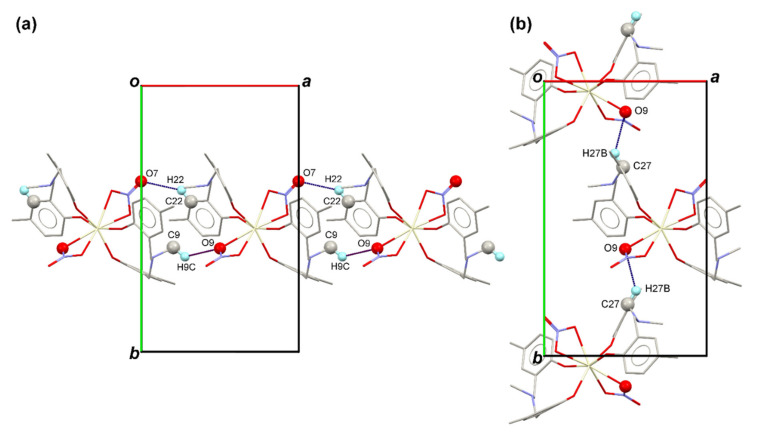
A [001] direction view showing (**a**) the part of molecular chain joined by the C9–H9C⋯O9 and C22–H22⋯O7 interactions propagating along the [100] direction and (**b**) the part of the molecular chain linked via the C27–H27B⋯O9 interactions propagating along the [010] direction.

In the crystal structure of the Ce-MMD complex, there are several interactions holding the molecules of the Ce-MMD complex together (Table 2). The first type of intermolecular interaction is C–H⋯O interactions. Since the molecules of the MMD ligands possess many methyl substituents, methylene groups, and benzene rings, the C–H bonds of those mentioned groups can interact with the oxygen atoms of the nitrate ligands in the Ce-MMD complex. For instance, the hydrogen of the benzene ring (H22) and the methyl group (H9C) are linked to the O7 and O9 atoms in the nitrate ligands, respectively. These C–H⋯O interactions (C22–H22⋯O7 and C9–H9C⋯O9) connect the molecules of the Ce-MMD complex into an infinite molecular chain that propagates along the [100] direction (Figure 3a). Moreover, the methylene hydrogen (H27B) is also linked with the O9 atom, setting up the infinite molecular chain that travels along the [010] direction (see Figure 3b).

**Figure 4:**
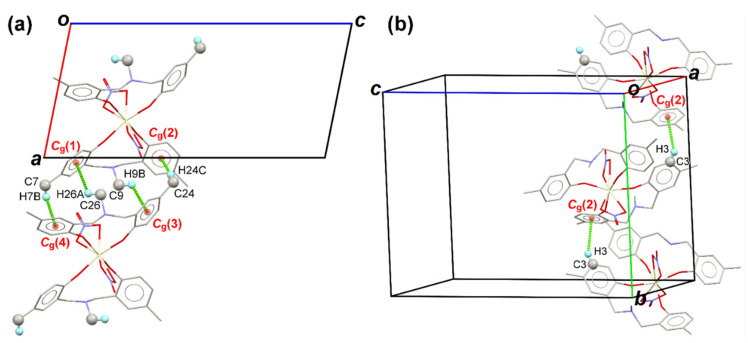
(**a**) A view down the [010] direction showing the part of the [100] molecular chain joined by several C_methyl_–H⋯π interactions, namely C26–H26A⋯*C*_g_(1), C24–H24C⋯*C*_g_(2), C9–H9B⋯*C*_g_(3), and C7–H7B⋯*C*_g_(4) contacts. (**b**) A view of a unit cell illustrating the part of the [010] molecular chain linked by the C3–H3⋯*C*_g_(2) interactions. Note that the *C*_g_(1), *C*_g_(2), *C*_g_(3), and *C*_g_(4) are the centroids constructed from the atoms C1–C6, C11–C16, C18–C23, and C28–C33, respectively.

**Figure 5:**
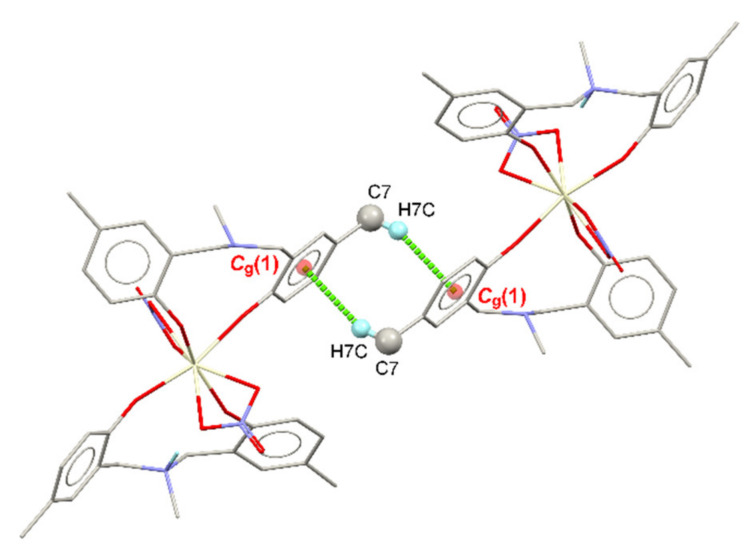
A view displaying the pair of C7–H7C⋯*C*_g_(1) contacts joining two molecules of the Ce-MMD complex related by an inversion symmetry.

Apart from the C–H⋯O interactions, numerous C–H⋯π contacts (see Figure 4) are also found in the crystal of the Ce-MMD complex due to the presence of several methyl groups and benzene rings. As depicted in Figure 4a, all the benzene rings are responsible for the formation of the C–H⋯π bonds between two molecules of the Ce-MMD complex. The molecules are located in the positions where the N-methyl groups are pointing towards the benzene rings, giving rise to the C26–H26A⋯*C*_g_(1) and C9–H9B⋯*C*_g_(3) interactions. The hydrogen atoms of methyl groups attached to the benzene rings are linked with the centroids of the benzene rings on another molecule, forming C24–H24C⋯*C*_g_(2) and C7–H7B⋯*C*_g_(4), implying that the single-molecule of the MMD ligand behaves as both the C–H⋯π donors and acceptors. These four mentioned C–H⋯π interactions link the molecules into an infinite molecular chain propagating along the [100] direction, which is the same direction (same symmetry code) as the C9–H9C⋯O9 and C22–H22⋯O7 interactions. Moreover, one more C_methyl_–H⋯π (C7–H7C⋯*C*_g_(1)) interaction is observed between two molecules related by the inversion symmetry (Figure 5). The C7–H7C⋯*C*_g_(1) contact has the H⋯*C*_g_ distance of 2.634 Å that is significantly shorter than the C_methyl_–H⋯π interactions mentioned earlier. Apart from the methyl groups, the benzene hydrogen can also build up the C–H⋯π (C3–H3⋯*C*_g_(2)) contact (Figure 4b). According to the presence of the various C–H⋯O and C–H⋯π interactions, the crystal structure of the Ce-MMD complex is very stable at room temperature. 

**Figure 6:**
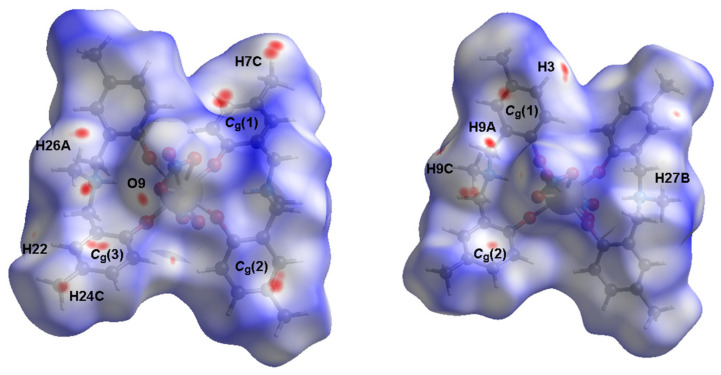
Two Hirshfeld surfaces viewing on the opposite sides mapped over *d*_norm_ in the range from −0.1157 to +1.5886 arbitrary units.

To visualize the essential interactions within the crystal of the Ce-MMD complex, Hirshfeld surfaces (HS) [63,64] were calculated by *Crystal Explorer 17.5* software [65]. Hirshfeld surfaces viewed on the opposite sides of the cerium-MMD molecules are illustrated in Figure 6. There are several faint red spots noticed in the HSs, which confirm most of the interactions discussed earlier. For example, the faint spots near H9C, H22, and H27B atoms confirm the presence of the C–H⋯O interactions. The occurrence of the C–H⋯π contacts can be readily seen from the red spots around the *C*_g_(1), *C*_g_(2), and *C*_g_(3) centroids. Note that no red spot is located around the *C*_g_(4) centroid since its C–H⋯π distance is longer than other C–H⋯π distances. There are some other spots situated on atoms that are not involved in the C–H⋯O and C–H⋯π interactions. This observation may be assigned to the C–H⋯C interactions found in the crystal structure. The HS study agrees well with the analysis of intermolecular interactions derived from the single crystal data, as discussed in the previous section.

**Figure 7:**
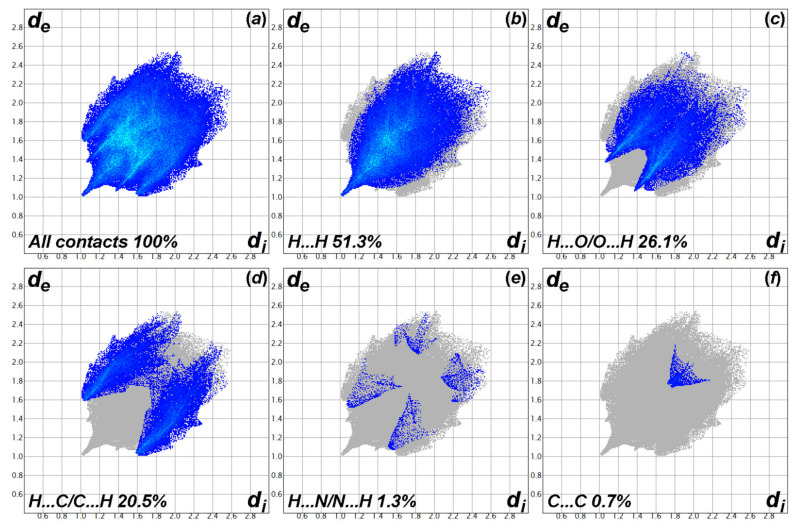
The two-dimensional fingerprint plots for the Ce-MMD complex, showing (**a**) all interactions, and those delineated into (**b**) H⋯H, (**c**) H⋯O/O⋯H, (**d**) H⋯C/C⋯H, (**e**) H⋯N/N⋯H, and (**f**) C⋯C contacts. The *d*_i_ and *d*_e_ values are the closest internal and external distances (in Å) from the given points on the Hirshfeld surfaces. The *d*_e_ + *d*_i_ values for the H⋯H, H⋯O/O⋯H, H⋯C/C⋯H, H⋯N/N⋯H, and C⋯C contacts are about 2.0, 2.45, 2.6, 2.6, 3.5 Å, respectively.

To obtain a quantitative contribution of each contact on the crystal packing, the corresponding 2D fingerprint plots are calculated [66]. The fingerprint plots showing all interactions, which are delineated into H⋯H, H⋯O/O⋯H, H⋯C/C⋯H, H⋯N/N⋯H, and C⋯C interactions, are demonstrated in Figure 7. According to the fingerprint plots, the contributions of the H⋯H, H⋯O/O⋯H, H⋯C/C⋯H, H⋯N/N⋯H, and C⋯C interactions are 51.3%, 26.1%, 20.5%, 1.3%, and 0.7%, respectively. This information reflects that the C–H⋯O and C–H⋯π interactions are the major contributors to the crystal packing of the Ce-MMD complex, since the combined contribution is 98.6%. Note that the H⋯N/N⋯H contact with the contribution of only 1.3% is due to the formation of the weak C–H⋯N interactions between the methylene hydrogen (H27B) and the nitrogen atom of the nitrate ligand (N4).

### 3.2. Spectroscopic Studies and Thermal Properties of the Ce-MMD Complex

**Figure 8:**
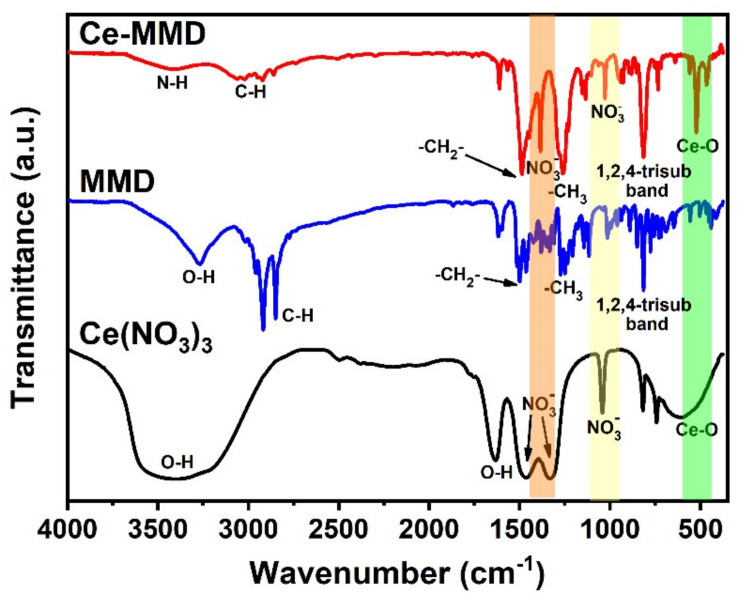
FTIR spectra of cerium(III) nitrate hexahydrate, the MMD ligand, and the Ce-MMD complex.

To further confirm the functional groups of the Ce-MMD complex, Fourier transform infrared (FTIR) spectroscopy was carried out. FTIR spectra of the Ce-MMD complex as well as its corresponding precursors, namely MMD ligand and cerium(III) nitrate hexahydrate, are illustrated in Figure 8. As seen from the FTIR spectra of the MMD and the Ce-MMD complex, it is clearly observed that the sharp O–H peak at 3271 cm^−1^ is not observed in the Ce-MMD complex, but the weaker peak at 3421 cm^−1^ is shown instead. This weaker peak is assigned to N–H stretching vibration regarding the R_3_NH ammonium group of the coordinated MMD ligand within the obtained complex. This suggests that the two phenolic hydrogens are deprotonated, while the tertiary amine group is protonated during the formation of the Ce-MMD complex. Two N–O peaks at 1330 and 1041 cm^−1^ are also found, revealing that the nitrate groups are involved within the complex structure. The peak positions of the N–O peaks in the Ce-MMD complex and the cerium(III) nitrate hexahydrate salt are very close to each other because the nitrate groups in both compounds are bonded to the Ce ion. The appearance of the sharp Ce–O stretching peak at 612 cm^−1^ confirms the coordination between the Ce ion and the oxygen atoms in both the nitrate and the coordinated (*N*-protonated and O,O-deprotonated) MMD ligands. This vibrational frequency is well-matched with the values reported in the literature [67]. Moreover, the characteristic peaks concerning the MMD ligand, such as CH_2_ bending at 1487 cm^−1^, CH_3_ bending at 1260 cm^−1^, and 1,2,4-trisubstituted band at 814 cm^−1^, are mainly retained in the Ce-MMD complex, in which some slight shifts in the peak positions are observed.

**Figure 9:**
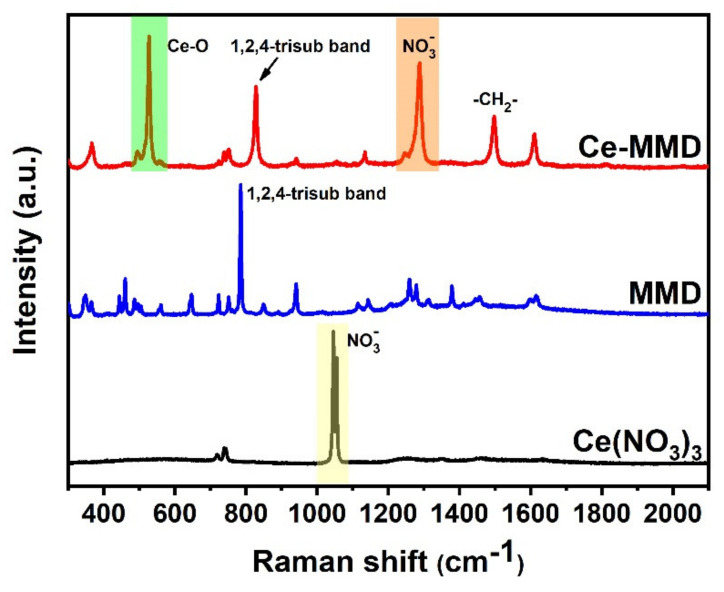
FT-Raman spectra of cerium(III) nitrate hexahydrate, the MMD ligand, and the Ce-MMD complex.

In addition, FT Raman spectroscopy is also performed (Figure 9). The number of peaks found in the FT Raman spectra is significantly less than those of the FTIR spectra, since the compounds studied herein possess certain symmetry elements. However, the observed peaks are enough to confirm the functional groups and chemical structures. For the Ce-MMD complex, the peaks at 1497, 1288, and 828 cm^−1^ are attributed to CH_2_ bending, N–O stretching, and the 1,2,4-trisubstituted band, respectively. Apart from FTIR and FT-Raman results, ^1^H-NMR data also support the structure of the Ce-MMD complex. For example, the O–H peak (δ 8.80) is diminished, while the N–H peak (δ 10.22) is additionally noticed, which indicates the protonation on the nitrogen atom and the deprotonation on the oxygen atoms of the coordinated MMD ligands after the complexation. All the mentioned observations strongly agree with the single-crystal X-ray crystallographic information discussed previously. 

**Figure 10:**
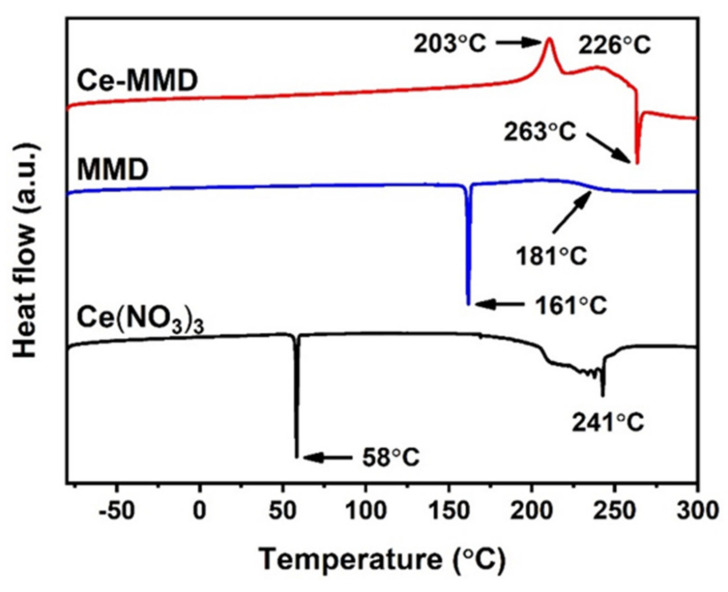
DSC thermograms of cerium(III) nitrate hexahydrate, the MMD ligand, and the Ce-MMD complex.

Thermal properties of the Ce-MMD complex and its starting materials were studied by differential scanning calorimetry (DSC), as shown in Figure 10. For the MMD ligand, a prominent endothermic peak was found at 161 °C, which is responsible for the melting point of the MMD ligand. The DSC thermogram of cerium(III) nitrate hexahydrate shows two endothermic peaks. The first sharp endothermic peak at 58 °C is assigned to the melting temperature of cerium nitrate hydrate. The second endothermic peaks in the range from 170 °C to 260 °C are due to the decomposition of the cerium nitrate. For the Ce-MMD complex, no evident peak is observed in the range from −80 to 200 °C. Two exothermic peaks are recognized after the temperature is higher than 200 °C. The first peak is observed as a sharp peak at 203 °C, and the second peak is a broader peak at 226 °C. These peaks are ascribed to the decomposition of the nitrate group when reacted with the organic ligands. The results highlight the oxidizing property of the nitrate group toward organic contents, giving out heat at elevated temperatures. As the decomposition progresses, heat energy is required, as the endothermic decomposition peak is noticed at approximately 263 °C. 

**Figure 11:**
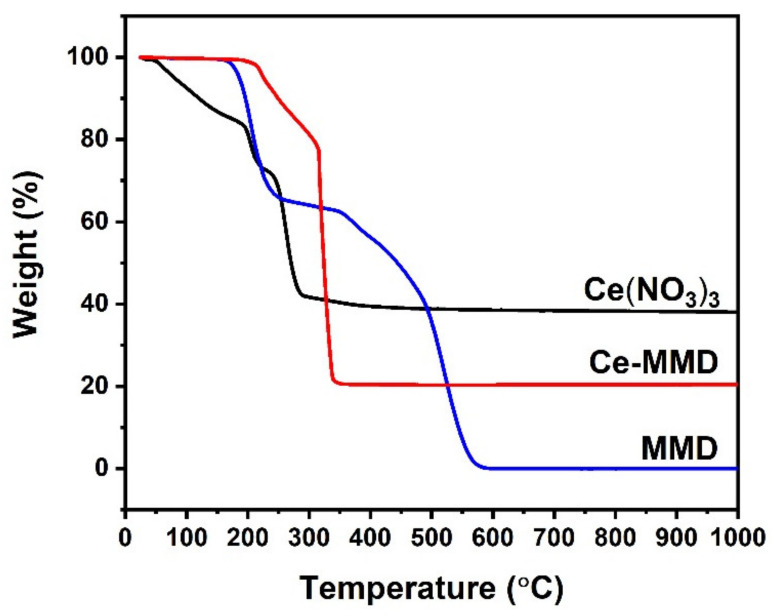
TGA thermograms of cerium(III) nitrate hexahydrate, the MMD ligand, and the Ce-MMD complex.

Moreover, thermogravimetric analysis (TGA) was also carried out to confirm the thermal events that occurred in the DSC study (Figure 11). For the MMD ligand, no weight loss is observed until the temperature reaches the onset temperature of 185 °C. The decomposition of the MMD ligand progresses until the end-set temperature of 556 °C. After the end-set temperature, the weight is decreased to about 0%, meaning that the MMD ligand had been reacted with oxygen gas in the air, forming only gaseous products such as CO, CO_2_, NO, NO_2_, and water vapor. Note that the water vapor and the NO_2_ gas might combine to form the HNO_3_ gas evolution [68]. The TGA thermogram of Ce(NO_3_)_3_·6H_2_O proceeds in two main stages, namely (1) dehydration to anhydrous cerium(III) nitrate and (2) the subsequent decomposition of the anhydrous cerium (III) nitrate to CeO_2_ by giving out NO_2_, NO, and O_2_ gases [69,70]. The onset temperature of the decomposition to CeO_2_ is found at 249 °C. This result is in agreement with the group of peaks at 170–260 °C observed in the DSC thermogram, indicating that the decomposition of anhydrous cerium(III) nitrate is the endothermic process. Moreover, the conversion from cerium(III) nitrate hexahydrate to ceria found during the heat treatment can be confirmed by the remaining weight of approximately 38% of the original weight. In the case of the Ce-MMD complex, the weight loss starts at a higher temperature than the cerium(III) nitrate hexahydrate, since it does not contain water within the inner and outer coordination sphere. The weight losses are seen after heating to 200 °C, which are corresponding to the DSC results. The first weight loss (200–315 °C) is assigned to the exothermic decomposition, while the second weight loss (315–327 °C) is attributed to the endothermic decomposition to obtain CeO_2_. Moreover, the weight remaining of about 20% strongly confirms the molecular formula (C_34_H_40_CeN_4_O_10_), purity, and homogeneity of the complex. Therefore, the DSC and TGA results are in line with the structure obtained from the SC-XRD technique.

### 3.3. Monitoring the Oxidative Complexation of the Cerium-MMD Complex

**Figure 12:**
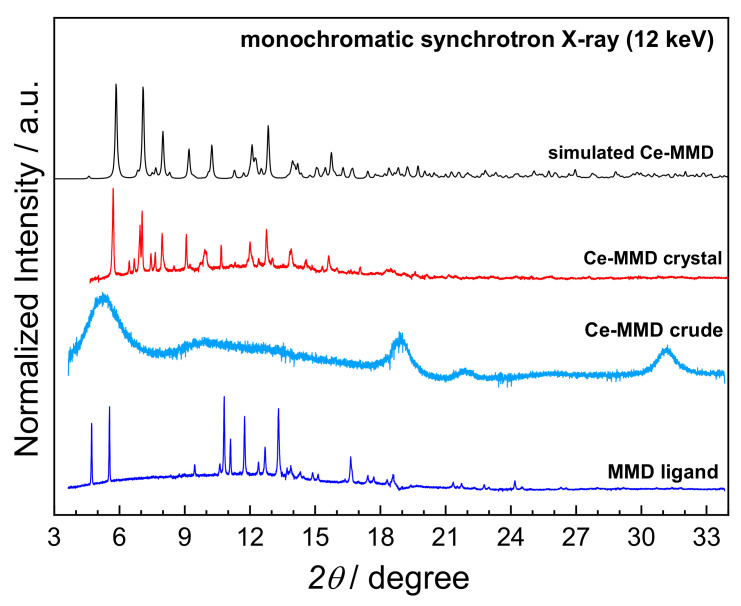
Comparison of powder X-ray diffraction (XRD) patterns of the MMD ligand (blue), the Ce-MMD crude (dark cyan), the Ce-MMD crystal (red), and the simulated XRD pattern of Ce-MMD based on the SC-XRD data (black).

Synchrotron powder X-ray diffraction (XRD) was used to characterize phase formation and purity of the obtained products at various steps of the synthetic procedure to understand the oxidative complexation formation of the Ce(IV)-MMD complex. According to the XRD patterns shown in Figure 12, the XRD pattern of Ce-MMD crystal is matched quite well with the simulated XRD pattern based on the single-crystal structure derived from SC-XRD, highlighting the high purity of the obtained product. There is also no crystalline impurity of the MMD ligand. Considering the Ce-MMD crude obtained by solvent evaporation from the ethanolic mixture between cerium(III) nitrate and MMD, a broad XRD pattern is observed. It indicates that the mixing of Ce(III) and MMD may immediately form the discrete Ce(III)-MMD complex dissolved in the ethanolic solution. The solvent evaporation directly after mixing the precursor solutions reduces the solubility and further leads to the formation of precipitations. However, it has only short- and medium-range periodic structural ordering, which consequently exhibits the broad XRD pattern.

**Figure 13:**
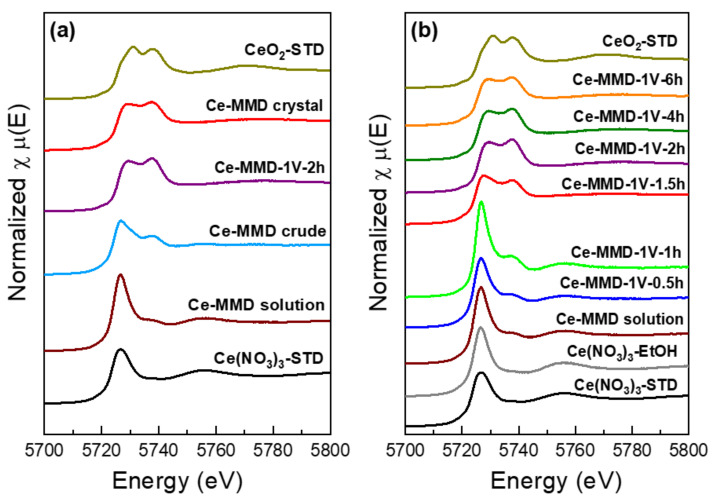
X-ray absorption near edge structure (XANES) spectra probed at Ce L3 edge to monitor the oxidative complexation formation of the desired Ce-MMD. (**a**) A comparison of Ce L3 edge XANES spectra of the obtained products at various stages of the synthetic procedure (namely the direct mixing Ce-MMD solution, the Ce-MMD crude, and the Ce-MMD crystal) with respect to the standard Ce(NO_3_)_3_ and CeO_2_ compounds. (**b**) The Ce L3 edge XANES spectra of the obtained products obtained from various stages during the chronoamperometry by applying a fixed voltage of 1 V to the mixture.

In addition, X-ray absorption spectroscopy (XAS) probed at the X-ray absorption near edge structure (XANES) region at the Ce L3 edge (see Figure 13) is carried out to verify the oxidation state and the chemical speciation of the obtained products at various stages of the synthetic procedure (namely the direct mixing Ce-MMD solution, the Ce-MMD crude, and the Ce-MMD crystal). The XANES spectra of the obtained products are compared to the standard Ce(NO_3_)_3_ and CeO_2_ compounds, as shown in Figure 13a. It is clearly seen that the direct mixing Ce-MMD solution exhibits the Ce(III) oxidation state and the chemical speciation of Ce(III)-MMD complex, of which the XANES spectrum is rather similar to the standard Ce(NO_3_)_3_. The deviation of the XANES spectrum is observed in the Ce-MMD crude, which indicates the combination between the Ce(III) and Ce(IV) chemical species. This implies that the solvent evaporation from the ethanolic solution containing Ce(III)-MMD complex induces the formation of the Ce(IV)-based complex. However, the oxidative complexation is not complete. The Ce-MMD crystal exhibits the Ce(IV) oxidation state and the chemical speciation of the Ce(IV)-MMD complex, of which the XANES spectrum is somewhat similar to the standard CeO_2_. 

The results from XRD and XAS reveal that the outer-sphere oxidation process of Ce(III) by oxygen in the air is gradually occurred to form the Ce(IV) center, to which the MMD and NO_3_^−^ ligands coordinate and consequently form the stable Ce(IV)-MMD crystal. In other words, the formation of Ce(IV)-MMD crystal takes place by the gradual oxidative complexation under ambient air conditions. Note that the prolongation of reaction time for at least two days is required to achieve the desired crystal. Therefore, the additional monitoring of the oxidative complexation of Ce-MMD by applying the voltage is carried out to let the complexation reaction occur faster.

Chronoamperometry using a conventional three-electrode system (a glassy carbon working electrode, a Pt wire counter electrode, and a silver/silver nitrate (0.01 M) reference electrode) is employed to apply a fixed voltage of 1 V to the ethanolic solution mixture between the cerium(III) nitrate hexahydrate and the MMD ligand. The obtained solutions and/or the precipitated powders after applying a static voltage of 1 V for 0.5 h, 1 h, 1.5 h, 2 h, 4 h, and 6 h together with the direct mixing of Ce-MMD solution are investigated by X-ray absorption spectroscopy probed at the Ce L3 edge, as shown in Figure 13b. The ethanolic Ce(NO_3_)_3_ solution and the direct mixing Ce-MMD solution exhibit a characteristic XANES feature of the Ce(III) species. By applying a voltage of 1 V for 0.5 and 1 h, no solid precipitation was observed. However, a slight deviation of the XANES feature from the common Ce(III) XANES spectra was observed in the obtained solutions, suggesting the initiation of the Ce(III) oxidation to form the Ce(IV) complex. A dark brown precipitate is firstly observed after applying a voltage of 1 V for 1.5 h. The prolongation of chronoamperometry for 2 h, 4 h, and 6 h also forms the dark brown precipitate. The obtained dark brown precipitates at different reaction stages are collected, and then the corresponding XANES spectra are measured. It is clearly shown that the Ce(IV)-MMD complex is the major product after applying a voltage of 1 V to the solution mixture for longer than 1.5 h. At least 2 h of reaction is required to obtain the complete oxidative complexation and form the desired Ce(IV)-MMD complex showing similar XANES spectra to the Ce(IV)-MMD single crystal. In all, the results indicate the outer-sphere oxidation process of Ce(III) by oxygen in the air and maybe also the oxidation-induced ligand redistributions to form the stable Ce(IV)-MMD-nitrate compound.

## 4. Conclusions

The single crystal of the novel Ce(IV)-MMD complex has been synthesized from the oxidative complexation between cerium(III) nitrate hexahydrate and MMD ligand under the ambient air. The crystallographic data obtained from single-crystal X-ray diffraction indicate the coordination of the Ce(IV) center by two nitrate ligands and two (*N*-protonated and O,O-deprotonated) MMD ligands in the complex. The coordinated MMD ligand binds to the Ce(IV) center in the bidentate fashion, utilizing two oxygen atoms of phenolate moieties to form the dative covalent bonds with the Ce(IV) center. A closer inspection into the fingerprint plots and the Hirshfeld surface analyses shows that the C–H⋯O and C–H⋯π interactions are the main contributors for the crystal packing, showing a total contribution of 98.6%, which link the molecules into infinite molecular chains propagating along the [100] and [010] directions. Moreover, it leads to the high stability of the desired complex at an ambient atmosphere up to 200 °C corresponding to the TGA and DSC thermograms. Synchrotron XRD and XAS studies propose the oxidative complexation to form the desired Ce(IV)-MMD complex via the outer-sphere oxidation process of Ce(III) by oxygen in the air and possibly the oxidation-induced ligand redistributions. Understanding the crystallographic information, the complex formation, and the corresponding physicochemical properties of the metal-organic complex could pave the way for rational designs of novel materials and provide an expansion toward the diversity of applications. The obtained Ce(IV)-MMD complex could be a potential candidate for further applications such as a well-controlled single-source precursor for oxide-based material syntheses via thermal decomposition, a redox catalyst, a photocatalyst, and a photoluminescent material. 

## Figures and Tables

**Table 1:** Crystal data and structure refinement details for the cerium-MMD complex.

Crystallographic Data and Structural Refinement Details	Cerium-MMD Complex
CCDC number	2098858
Empirical formula	C_34_H_40_CeN_4_O_10_
Formula weight	804.82
Temperature/K	100
Crystal system	Monoclinic
Space group	P2_1_/c
a/Å	10.0752(18)
b/Å	16.710(3)
c/Å	20.660(4)
α/°	90
β/°	101.317(7)
γ/°	90
Volume/Å^3^	3410.6(11)
Z	4
ρ_calc_g/cm^3^	1.567
μ/mm^−1^	1.398
F(000)	1640.0
Crystal size/mm^3^	0.2 × 0.14 × 0.1
Radiation	MoK_α_ (λ = 0.71073)
2Θ range for data collection/°	3.16 to 50.556
Index ranges	−12 ≤ h ≤ 12, −20 ≤ k ≤ 14, −23 ≤ l ≤ 24
Reflections collected	18,342
Independent reflections	6132 [R_int_ = 0.0867, R_sigma_ = 0.1083]
Data/restraints/parameters	6132/0/448
Goodness-of-fit on F^2^	1.055
Final R indexes [I ≥ 2σ (I)]	R_1_ = 0.0640, wR_2_ = 0.1431
Final R indexes [all data]	R_1_ = 0.1017, wR_2_ = 0.1618
Largest diff. peak/hole/e Å^−3^	2.05/−1.90

**Table 2:** Geometries of N–H⋯O, C–H⋯O, and C–H⋯π interactions (Å, °).

D–H⋯A	d(D–H)/Å	d(H⋯A)/Å	d(D⋯A)/Å	D–H⋯A/°
N–H⋯O interactions				
N1–H1⋯O1	0.98	1.92	2.717(8)	136
N1–H1⋯O2	0.98	2.40	3.056(8)	124
N2–H2⋯O3	0.98	1.98	2.753(8)	134
N2–H2⋯O4	0.98	2.41	3.061(8)	123
C–H⋯O interactions				
C9–H9C⋯O9 ^i^	0.96	2.541	3.242	130
C22–H22⋯O7 ^i^	0.93	2.660	3.421	140
C27–H27B⋯O9 ^ii^	0.97	2.672	3.417	134
C–H⋯π interactions				
C26–H26A⋯*C*_g_(1) ^i^	0.96	2.908	3.502	121
C24–H24C⋯*C*_g_(2) ^i^	0.96	2.840	3.790	171
C9–H9B⋯*C*_g_(3) ^i^	0.96	2.864	3.423	118
C7–H7B⋯*C*_g_(4) ^i^	0.96	3.015	3.481	111
C3–H3⋯*C*_g_(2) ^iii^	0.93	2.920	3.727	146
C7–H7C⋯*C*_g_(1) ^iv^	0.96	2.634	3.543	158

Symmetry codes: (i) −1 + x, y, z, (ii) 1 − x, −1/2 + y, ½ − z, (iii) 2 − x, 1 − y, z, (iv) 2 − x, − 1/2 + y, ½ − z.

## Data Availability

Crystallographic data have been deposited at the Cambridge Crystallographic Data Centre under the reference number CCDC 2098858.

## Sample Availability

Samples of the compounds regarding this article are available from the authors.
